# Longitudinal Analysis of Brain-Predicted Age in Amnestic and Non-amnestic Sporadic Early-Onset Alzheimer's Disease

**DOI:** 10.3389/fnagi.2021.729635

**Published:** 2021-11-03

**Authors:** Morgan Gautherot, Grégory Kuchcinski, Cécile Bordier, Adeline Rollin Sillaire, Xavier Delbeuck, Mélanie Leroy, Xavier Leclerc, Jean-Pierre Pruvo, Florence Pasquier, Renaud Lopes

**Affiliations:** ^1^UMS 2014–US 41–PLBS–Plateformes Lilloises en Biologie & Santé, University of Lille, Lille, France; ^2^Inserm, U1172–LilNCog–Lille Neuroscience & Cognition, University of Lille, Lille, France; ^3^Neuroradiology Department, Lille University Medical Centre, Lille, France; ^4^Memory Center, DISTALZ, Lille, France; ^5^Neurology Department, Lille University Medical Centre, Lille, France

**Keywords:** brain age, deep learning, structural MRI, longitudinal analysis, early-onset Alzheimer's disease, phenotypic variants

## Abstract

**Objective:** Predicted age difference (PAD) is a score computed by subtracting chronological age from “brain” age, which is estimated using neuroimaging data. The goal of this study was to evaluate the PAD as a marker of phenotypic heterogeneity and severity among early-onset Alzheimer's disease (EOAD) patients.

**Methods:** We first used 3D T1-weighted (3D-T1) magnetic resonance images (MRI) of 3,227 healthy subjects aged between 18 and 85 years to train, optimize, and evaluate the brain age model. A total of 123 participants who met the criteria for early-onset (<65 years) sporadic form of probable Alzheimer's disease (AD) and presented with two distinctive clinical presentations [an amnestic form (*n* = 74) and a non-amnestic form (*n* = 49)] were included at baseline and followed-up for a maximum period of 4 years. All the participants underwent a work-up at baseline and every year during the follow-up period, which included clinical examination, neuropsychological testing and genotyping, and structural MRI. In addition, cerebrospinal fluid biomarker assay was recorded at baseline. PAD score was calculated by applying brain age model to 3D-T1 images of the EOAD patients and healthy controls, who were matched based on age and sex. At baseline, between-group differences for neuropsychological and PAD scores were assessed using linear models. Regarding longitudinal analysis of neuropsychological and PAD scores, differences between amnestic and non-amnestic participants were analyzed using linear mixed-effects modeling.

**Results:** PAD score was significantly higher for non-amnestic patients (2.35 ± 0.91) when compared to amnestic patients (2.09 ± 0.74) and controls (0.00 ± 1). Moreover, PAD score was linearly correlated with the Mini-Mental State Examination (MMSE) and the Clinical Dementia Rating Sum of Boxes (CDR-SB), for both amnestic and non-amnestic sporadic forms. Longitudinal analyses showed that the gradual development of the disease in patients was accompanied by a significant increase in PAD score over time, for both amnestic and non-amnestic patients.

**Conclusion:** PAD score was able to separate amnestic and non-amnestic sporadic forms. Regardless of the clinical presentation, as PAD score was a way of quantifying an early brain age acceleration, it was an appropriate method to detect the development of AD and follow the evolution of the disease as a marker of severity as MMSE and CDR-SB.

## Introduction

Throughout life, the brain develops and changes (Teissier et al., 2020). The changes do not occur to the same extent in all brain regions (Trollor and Valenzuela, 2001) and are not uniform over the ages (Scahill et al., 2003). Brain aging does not only impact the function of our brain, it also impacts the structures with a decrease in white matter (WM) and gray matter (GM), and an increase in cerebrospinal fluid (CSF) brain volumes in adulthood (Guttmann et al., 1998). In contrast to WM, the volume decrease in GM is less uniform, with the frontoparietal cortex being more affected than the temporo-occipital cortex (Resnick et al., 2003). However, the shrinkage does not necessarily result from a decrease in the number of neurons but mainly from a reduction in their volume (Dickstein et al., 2007). Therefore, normal cellular brain aging is characterized more by subtle changes than a large-scale loss of cells (Teissier et al., 2020). As a result, it is more difficult to characterize the pace of these changes, the biological age of the brain, and all the processes involved in brain aging (Peters, 2006).

It is now widely assumed that Alzheimer's disease (AD) reflects a form of accelerated aging (Cao et al., 2010; Jones et al., 2011; Saetre et al., 2011). For this reason, a growing number of studies investigated both normal and AD age-related changes (Raji et al., 2009; Beheshti et al., 2020). Brain region volumetry may be of interest in the diagnosis of AD with a relatively preserved prefrontal cortex region and an atrophy of hippocampus compared to healthy people (Head et al., 2005; Jack and Holtzman, 2013). However, significant phenotypic heterogeneity of AD is widely recognized, as several atypical variants are described other than the typical limbic-predominant subtype, which is characterized by an amnestic presentation and a pattern of brain atrophy preferentially localized to the limbic areas (Ferreira et al., 2020). Atypical variants are characterized by a hippocampal-sparing pattern of brain atrophy that relatively spares the limbic structures but more severely affects neocortical areas (Whitwell et al., 2012; Cho et al., 2013; Risacher et al., 2017). Moreover, there is significant heterogeneity in the locations of atrophy across individual patients (Tetreault et al., 2020).

Magnetic resonance imaging (MRI) is a powerful non-invasive tool to investigate brain structural changes throughout ages *in vivo* (Guttmann et al., 1998; Sowell et al., 2003). These images can show changes in GM and WM during the maturation of the brain (Giedd et al., 1999; Paus, 1999; Sowell et al., 1999, 2001; Courchesne et al., 2000; Thompson et al., 2000) and during aging (Bartzokis et al., 2001; Jernigan et al., 2001). Many markers, such as cortical thickness and volumetric measures, are associated with brain aging in healthy controls and neurodegenerative diseases (Raji et al., 2009). Although the brain undergoes characteristic changes due to aging over the course of a lifetime, the impact may be slightly different for each individual. Not only are structural characteristics involved in brain change but also education and occupation may be proxies for brain functional reserve, reducing the severity and delaying the clinical expression of AD pathology.

Deep learning techniques, such as convolutional neural networks (CNN), have the benefits to identify MRI markers, and they can model complex non-linear relationships without the need for predefined traditional MRI markers (Cole et al., 2017b; Beheshti et al., 2018). However, these models need large and diverse samples for training the complex deep network, making it possible only by several data sharing initiatives. A growing field of research combining MRI markers and CNN algorithms are focusing on brain age estimation in the healthy population (Sajedi and Pardakhti, 2019), with a mean absolute error (MAE) of 3–5 years in age ranging from 18- to 90-year-olds using T1-weighted (T1w) structural MRI (Cole et al., 2017a; Franke and Gaser, 2019; Couvy-Duchesne et al., 2020). Predicted age difference (PAD), defined as the difference between chronological age and predicted age, is associated with disease status, including AD and mild cognitive impairment (Franke et al., 2010; Franke and Gaser, 2012; Löwe et al., 2016).

Early-onset AD (EOAD), which is defined by an age of onset ≤ 65 years, is of interest in the study of the phenotypic heterogeneity due to the higher frequency of non-amnestic variants than in late-onset AD (LOAD) (Palasí et al., 2015). Atypical presentations affect language abilities, visuospatial abilities, or executive functions (Marshall et al., 2007; Garre-Olmo et al., 2010; Koedam et al., 2010; Balasa et al., 2011; Sá et al., 2012). Patients with EOAD appear to exhibit faster cognitive decline than patients with LOAD (Haxby et al., 1992; Pettigrew et al., 2017). However, studies on LOAD tend to show that the later onset of dementia was the only prominent variable accelerating all cognitive and functional outcomes (de Oliveira et al., 2018). Moreover, studies on cognitive reserve tend to show the same result. The concept of cognitive reserve arose from the idea that life experiences associated with cognitive stimulation could increase brain resilience to neuropathologic lesion and delay the onset of symptoms of functional decline (Haxby et al., 1992; Soldan et al., 2020). However, cognitive reserve did not have a linear effect on the development of brain injuries, and even experienced a paradox. Previous studies suggest that while the cognitive reserve is associated with a delayed symptom onset (Qiu et al., 2001; Reed et al., 2010; Roe, 2011; Soldan et al., 2013; Pettigrew et al., 2017; Robitaille et al., 2018; van Loenhoud et al., 2019), it could be related to more severe brain atrophy and accelerated cognitive decline in advanced AD stages (Wilson et al., 2000; Scarmeas, 2005; Andel et al., 2006; Bracco et al., 2007; Hall et al., 2007; Yoon et al., 2015; Myung et al., 2016; Soldan et al., 2017). These contradictory results may be explained by a higher proportion of non-amnestic variants and higher education level (which seems to be a proxy for cognitive reserve) in EOAD patients.

Despite the growing interest to better understand the mechanisms underlying phenotypic heterogeneity in EOAD using MRI (Mendez, 2012; Falgàs et al., 2020a; Vanhoutte et al., 2020), there is a potential bridge between these group-level studies and the clinical care of individual AD patients. The aim of this study was to investigate the PAD as a marker of phenotypic heterogeneity in EOAD for diagnostic and follow-up purposes. We hypothesized that (i) PAD marker would distinguish between clinical variants of EOAD, and (ii) progression of PAD marker would follow the functional and cognitive severity of disease for both phenotypes.

## Materials and Methods

### EOAD Population

Participants with EOAD were all recruited at the University Hospital's Memory Resources and Research Center in Lille, France. The participants were part of the COhorte Malade Alzheimer's Jeunes (Early-onset Alzheimer's cohort in French, COMAJ), which was initiated in 2009.

The COMAJ study was approved by the local institutional investigational review board [Ethic committee (CPP Nord-Ouest I); reference: 110-05]. Written informed consent was obtained from all participants and/or their relatives. Inclusion criteria were as follows: (a) participants should meet National Institute on Aging - Alzheimer's Association (NIA-AA) criteria for “probable AD dementia with intermediate evidence of AD patho-physiological process” (McKhann et al., 2011) and International Working Group (IWG) 2 criteria (Dubois et al., 2014); (b) participants must be ≤ 60 years of age at the time of first symptoms; (c) evidence of abnormal CSF biomarkers with Aβ_42_ below 700 pg/mL and total tau and phosphorylated tau above 400 and 60 pg/mL, respectively (Lehmann et al., 2014). The final diagnosis of sporadic EOAD was based upon extensive reviewing of clinical history, CSF biomarkers, and neuropsychological and imaging data by a multidisciplinary board. Criteria for pathological mutations were onset of symptoms <51 years old or family history of EOAD in the first degree. Individuals with early-onset dementia in first-degree relatives or those with a confirmed mutation in the PSEN1, PSEN2, or APP genes were excluded. Out of 123 participants, 16 participants were searched for APP, PSEN1, and PSEN2.

A total of 217 sporadic EOAD participants were included and classified as amnestic presentation (“typical”) or non-amnestic presentation (“atypical”) with prominent cognitive impairments in language, visuospatial, or executive functions, based on neuropsychological tests of 4 cognitive domains listed below:

1. Episodic memory: free and cued selective recall, the “doors” part of the Doors and People test, and the Visual Association Test (Lindeboom, 2002; Schoonenboom et al., 2005).
2. Language: the DO80 confrontation naming test with 80 images (Deloche and Hannequin, 1997).
3. Visuospatial function: evaluation of upper limb praxis, evaluation of visuoconstructive abilities using the Rey-Osterrieth complex figure test and the Beery-Buktenica developmental test of visual-motor integration (Beery VMI test) (Lim et al., 2015), evaluation of visual gnosis using subtests from the Visual Object and Space Perception Battery (the screening test, incomplete letters, and number location).
4. Executive functions: evaluation of working memory using the forward and backward digit span task from the Wechsler memory scale (third edition), the Frontal Assessment Battery at bedside, category verbal fluency (animals), and lexical verbal fluency (the letter P) (Godefroy, 2008).

**Figure 1:**
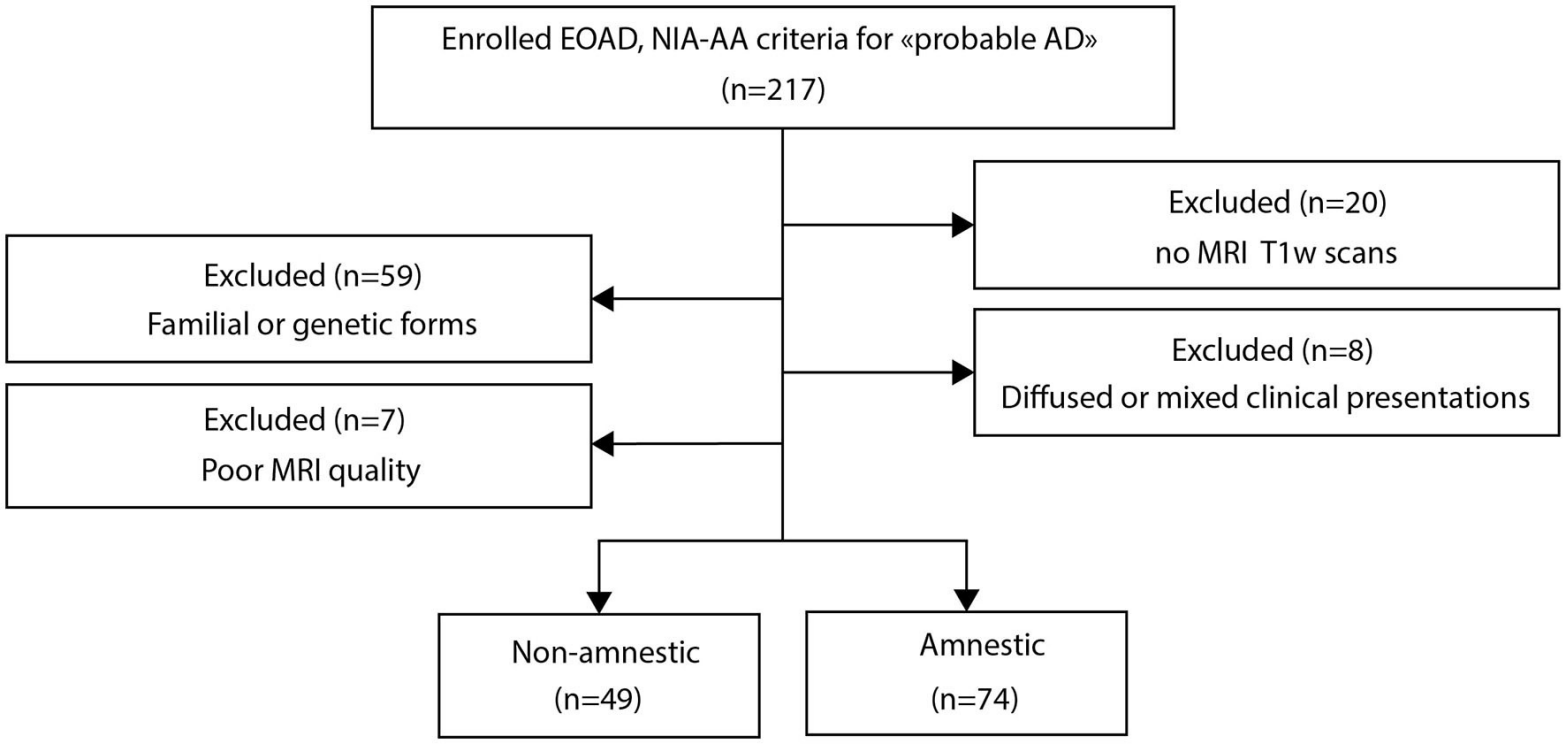
Flowchart of the study population.

Three-dimensional T1-weighted (3D-T1) images were acquired on a 3T MRI scanner (Achieva, Philips, Best, the Netherlands), using an 8-channel phased-array head coil and whole-body coil transmission (field of view = 256 × 256 × 160 mm^3^, isotropic voxel size 1 × 1 × 1 mm^3^, TR = 9.9 ms, TE = 4.6 ms, and flip angle = 8°) for initial evaluation and follow-up. In addition to MRI, each patient was evaluated annually for a maximum of 4 years by the Mini-Mental State Examination (MMSE) (Folstein et al., 1985) and the Clinical Dementia Rating Sum of Boxes (CDR-SB) (Hughes et al., 1982). Predicted brain age was obtained from participants who completed a structural MRI scan at least for one time point during follow-up. Based on the availability and quality of MR images of the 217 EOAD participants, 74 amnestic and 49 non-amnestic participants were finally retained (Figure 1). Out of the total number, 70, 28, 21, 17, 8 amnestic and 46, 49, 38, 28, 13 non-amnestic patients were finally retained at baseline, year 1, year 2, year 3, and year 4, respectively (Supplementary Table S1).

### Healthy Population

A total of 3,227 MRI scans from 2,065 healthy participants (48% men, mean age = 33.6 ± 12.3 years, age range from 18 to 85 years) were included in the study. Data were compiled from publicly available sources made available *via* various data sharing initiatives (Supplementary Table S2). According to the local study protocols, all participants were free from neurological or psychiatric disease. We retained only images acquired at 3T MRI using 3D-T1 sequence. The subject consent was obtained at each local study site and each contribution was ethically approved.

These images were divided into three datasets—a control dataset to compare with the patients from the COMAJ cohort, the training dataset to train and optimize our model, and the testing dataset to test its performance.

For our control dataset, we used images of 116 age- and sex-matched healthy subjects from the Parkinson's Progression Markers Initiative (PPMI) (www.ppmi-info.org/data) and the Alzheimer's Disease Neuroimaging Initiative (ADNI) (adni.loni.usc.edu). The ADNI was launched in 2003 as a public–private partnership, led by Principal Investigator, Michael W. Weiner, MD. The primary goal of ADNI has been to test whether serial MRI, positron emission tomography and other biological markers, and clinical and neuropsychological assessment can be combined to measure the progression of mild cognitive impairment and early AD. These images were acquired by scanners from the three major MRI manufacturers (General Electric, Philips, and Siemens). These data were never used in the training, the hyperparametrization, or the testing of our brain age model.

The training and testing sets were composed of 3,083 MR acquisitions from 1,955 subjects and 144 MR acquisitions from 110 subjects obtained from 6 data sharing initiatives, which include Information eXtraction from Images (IXI), Human Connectome Project (HCP) (Van Essen et al., 2012), Center Of Biomedical Research Excellence (COBRE), Mind Clinical Imaging Consortium (MCIC), Neuromorphometry by Computer Algorithm Chicago (NMorphCH), and enhanced Nathan Kline Institute-Rockland Sample (NKI-RS) (more details in Supplementary Table S2).

### Data Preprocessing

Minimal preprocessing steps were performed on 3D-T1 images (Lombardi et al., 2020). First, images were corrected for magnetic field inhomogeneity effects and skull-stripped using VolBrain software (volbrain.upv.es) (Manjón and Coupé, 2016). Brain extractions were systematically checked for possible errors (brain regions missing), and manual corrections were performed by a neuroradiologist (GK), when deemed necessary (Fischl, 2012). Then, preprocessed 3D-T1 images were linearly registered into MNI space and resampled to 1 mm^3^ using SPM software (fil.ion.ucl.ac.uk/spm/software/spm12). Finally, intensity normalization was performed using min–max normalization.

Furthermore, for correlation purposes, GM, WM, and CSF brain volumes were estimated using VolBrain software (Manjón and Coupé, 2016).

### Brain Age Prediction Model

**Figure 2:**
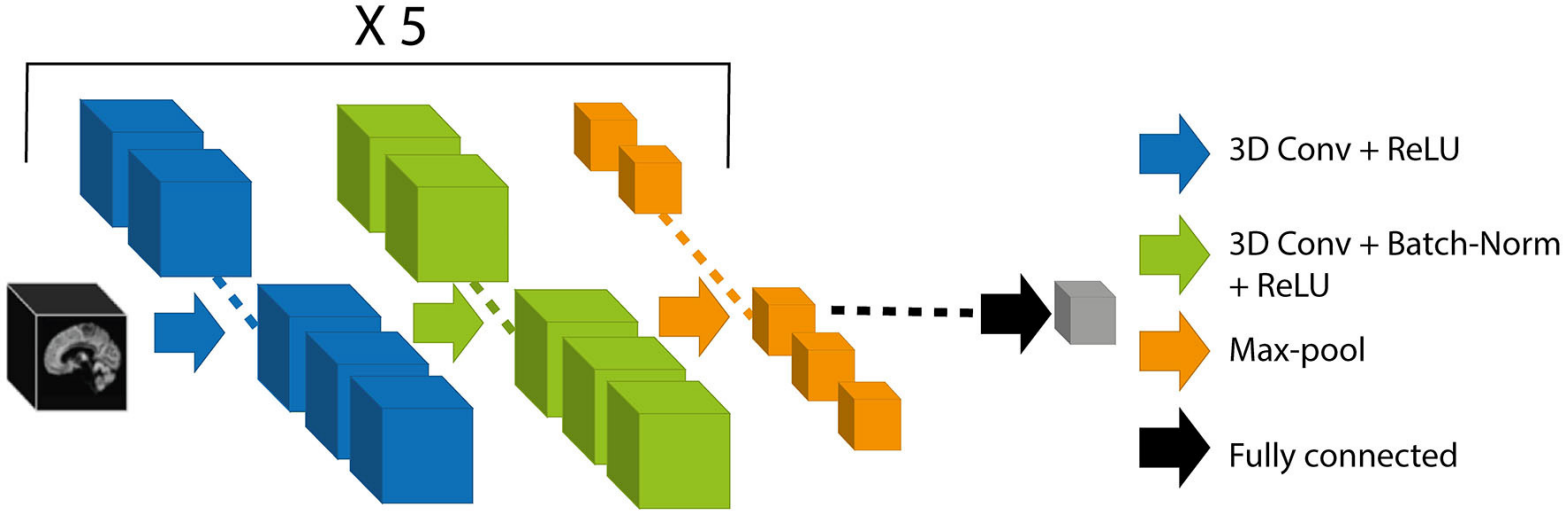
Schematic representation of the CNN architecture. The black box represents the 3D-T1 images as inputs of our model. Each block consisted of 3D convolution of kernel size (3 × 3 × 3), ReLU activation, 3D convolution of kernel size (3 × 3 × 3), batch normalization, rectified linear unit activation, and max-pooling layer of pooling size (2 × 2 × 2). The block was repeated 5 times. The output of the network (gray box) corresponds to the brain-predicted age. CNN, convolutional neural network; ReLU, Rectified Linear Unit.

For the prediction of chronological age using MRI from healthy control subjects, also called “brain age,” our model was based on 3D convolutional neural network (CNN) architecture. This architecture, which was inspired by Cole et al. (2017a), is both simple and efficient for the prediction of brain age using 3D-T1 images (Figure 2).

The proposed architecture took preprocessed 3D-T1 images with dimensions of 182 × 218 × 182 voxels. The weights of the model were determined by minimizing the cost function, here the mean absolute error (MAE). To optimize the weights, we used stochastic gradient descent optimization algorithm (Sutskever et al., 2013) with a learning rate of 0.001, a momentum of 0.1, and a learning rate decay of 5e-05. We used a batch size of 8 during 150 iterations. We performed an early stopping at the epoch 113 because it gave us the best MAE on the validation set.

During the training phase, we performed a data augmentation strategy on-the-fly consisting of performing translation and rotation of the MR images. This technique generated additional artificial training images to prevent the model from overfitting and was empirically found to yield better performance (Shorten and Khoshgoftaar, 2019).

**Figure 3:**
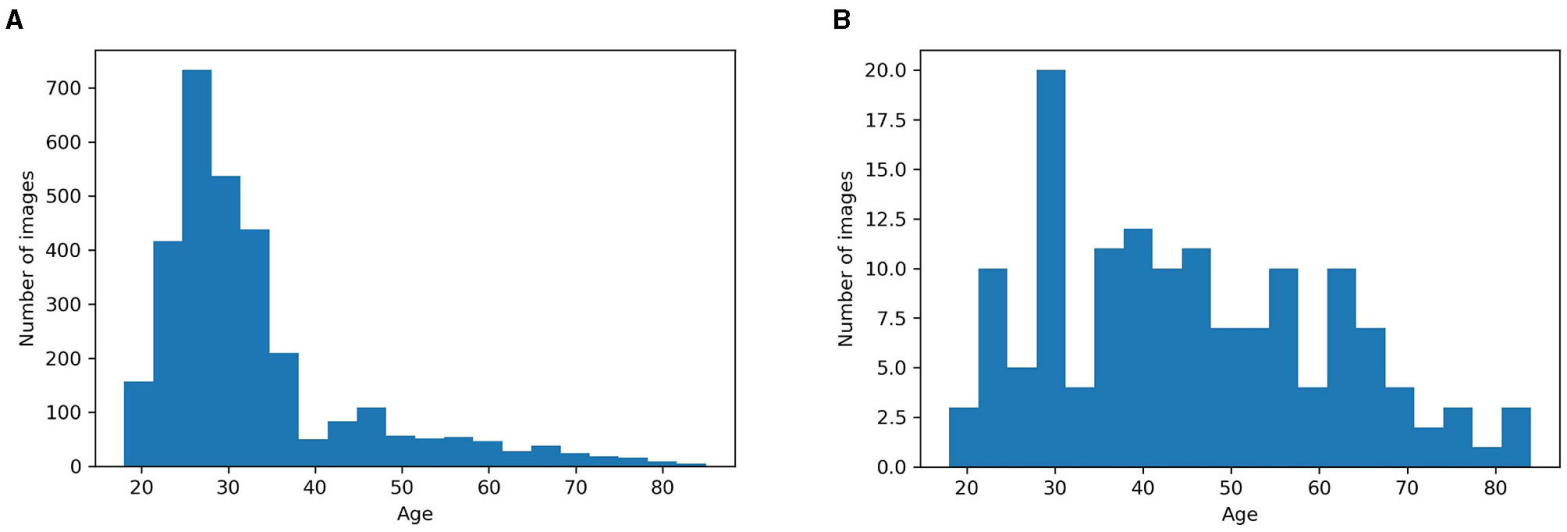
**(A)** Age distribution of subjects in the training set. **(B)** Age distribution of subjects in our testing set. We randomly selected 10 images every 5 years to obtain a uniform distribution of images throughout the age.

We used a 5-fold cross-validation procedure on our training set for optimizing hyperparameters and for assessing how our results would generalize to another dataset of the same distribution. The distribution of patient ages was not uniform in the training set, so in the cross-validation, the distribution of the validation set was not uniform either (Figure 3A).

To test the performance of our model across the ages, we needed a more balanced testing set. The testing set had 144 images from 110 subjects, with 10 randomly selected images for each 5 years period from 18 to 70 years, and 10 randomly selected images from 70 years and above. With this pseudo-random selection, we obtained a more uniform distribution than the training age distribution (Figure 3B).

### Bias Correction in Brain Age Prediction

Like all regression methods, the brain age model is subject to the fundamental phenomenon of “regression toward the mean” (Galton, 1886). This bias overestimates age among younger participants and underestimates it in older participants. Although studies had mainly attributed the bias to inconsistency in the distribution of noise over the life course (Cole et al., 2017a), the reasons are still largely unknown. The bias seems rather universal, regardless of the data, age range, sample size, and even the particular methods used (linear machine learning or deep learning methods) (Liang et al., 2019). To correct the regression toward the mean phenomenon, we used the following equation (Liang et al., 2019):


(1) $$\begin{array}{r} regressed\;age\;predicted=intercept+\alpha^{*}chronological\;age\;+\\ \;error \end{array}$$

α is a regression coefficient associated with the chronological age, and in our study α = 0.13.

### Patient Prediction

Weights from the training model were used for the prediction of brain age of healthy controls and EOAD patients. This age was regressed out using Equation 1. PAD score was calculated as the difference between predicted brain age and chronological age at the acquisition time. We calculated the PAD z-score for the three groups taking the control group as standard.

### Model Visualization With Gradient Class Activation Maps

Gradient Class Activation Maps (Grad-CAM) approach uses the final convolutional layer gradients to produce a map highlighting the brain regions used for brain age prediction (Selvaraju et al., 2017). Grad-CAM approach was used to create an average map called an attention map for each group of participants (controls, non-amnestic EOAD, and amnestic EOAD participants).

### Statistical Analysis

All the analyses were conducted in Python (3.8.5) using scipy (1.6.1) and statsmodels (0.12.1). At baseline, intergroup differences between controls, amnestic, and non-amnestic EOAD participants in demographic, clinical, and neuropsychological features were assessed using Wilcoxon or Kruskal–Wallis tests for continuous variables and chi-squared tests for categorical variables. To quantify the magnitude of effect sizes between groups, we used Cliff's delta (Cliff, 1993). Between-group differences for neuropsychological and PAD scores were assessed using linear models. Regarding the longitudinal analysis of neuropsychological and PAD scores, differences between amnestic and non-amnestic participants were analyzed using univariate linear mixed-effects (LME) models. LME models provide an approach for analyzing longitudinal data while handling variable missing rates and non-uniform timing. These models also make use of participants with a single time point to characterize population-level regionally specific differences. Equality of regression coefficients was assessed by the Chow test (Toyoda, 1974).

The threshold for statistical significance was set to *p* < 0.05. Bonferroni *post-hoc* test was used to correct for multiple comparisons.

### Code Availability

Code is available on github at https://github.com/MorganGautherot/Brain_age_model.

## Results

### Demographic and Clinical Data at Baseline

Baseline demographic and clinical data between amnestic and non-amnestic EOAD forms are shown in Table 1. The non-amnestic and amnestic EOAD groups did not differ significantly with regard to age at inclusion, disease duration from the first symptoms, and cognition (CDR-SB and MMSE). The only difference was the education level, which was higher for the non-amnestic patients (10.41 ± 3.68) when compared with the amnestic patients (9.64 ± 2.82). Overall, the EOAD participants had a moderately severe disease (MMSE score was 17.17 ± 6.71 for amnestic, and 16.13 ± 6.96 for non-amnestic patients). Thus, education level was used as a covariate in the comparison of EOAD groups.

**Table 1:** Demographic and clinical characteristics at baseline according to clinical forms of EOAD and controls.

**Indicators**	**Amnestic**	**Non-amnestic**	**Control**	**Effect/*p*-value**
*N*	70	46	116	
CDR-SB	6.71 ± 4.25	7.21 ± 3.81		0.327^a^
MMSE	17.14 ± 6.71	16.13 ± 6.96		0.192^a^
Disease duration, years	5.45 ± 2.91	4.55 ± 2.14		0.157^a^
Education level, years	9.64 ± 2.82	10.41 ± 2.82		0.0357^a^^c^
Age, years	59.30 ± 4.28	58.61 ± 3.68	59.05 ± 4.05	0.611^b^
Female, n (%)	36 (51%)	26 (56%)	63 (54%)	0.73^a^

*Quantitative variables were quoted as the mean ± SD (interquartile range)*.

a *χ^2^ and Wilcoxon tests were applied to categorical and continuous variables, respectively*.

b *Kruskal–Wallis test was applied to continuous variables for more than two populations*.

c *p <0.05*.

*CDR-SB, Clinical Dementia Rating Sum of Boxes; MMSE, Mini-Mental State Examination*.

### Convolutional Neural Networks Accurately Predict Age Using Neuroimaging Data

**Figure 4:**
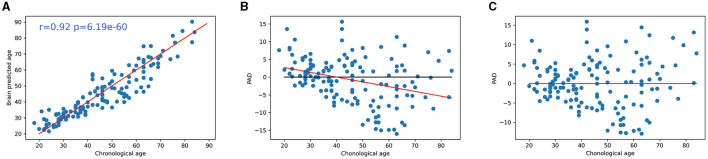
**(A)** Accuracy of CNN for brain age prediction using testing set. Scatter plots depict chronological age (x-axis) and brain-predicted age (y-axis) on the testing set subjects. The r value was the Pearson's correlation coefficient of brain-predicted age with chronological age and p was the associated *p*-value. Plot of the chronological age in function to PAD z-score **(B)** before and **(C)** after regression. CNN, convolutional neural network; PAD, predicted age difference.

Analysis showed that our CNN model accurately predicted the chronological age of healthy subjects, using 3D-T1 images. We obtained an MAE of 3.18 ± 0.43 years on training data, after the cross-validation approach (Table 2), and 4.34 years on the testing set (Figure 4A).

**Table 2:** Model performance on cross-validation datasets.

**Folds**	**1**	**2**	**3**	**4**	**5**	**Mean ± SD**
MAE validation (year)	3.85	2.74	3.06	2.77	3.48	3.18 ± 0.43

*MAE, mean absolute error; SD, standard deviation*.

Significant correlation was found between age and prediction error before bias correction (*r* = −0.33, *p* < 0.001), but not after bias correction (*r* = 0.00, *p* = 0.99) (Figures 4B,C).

### Comparison of Predicted Brain Age Between Participants of EOAD Subtypes and Controls

**Figure 5:**
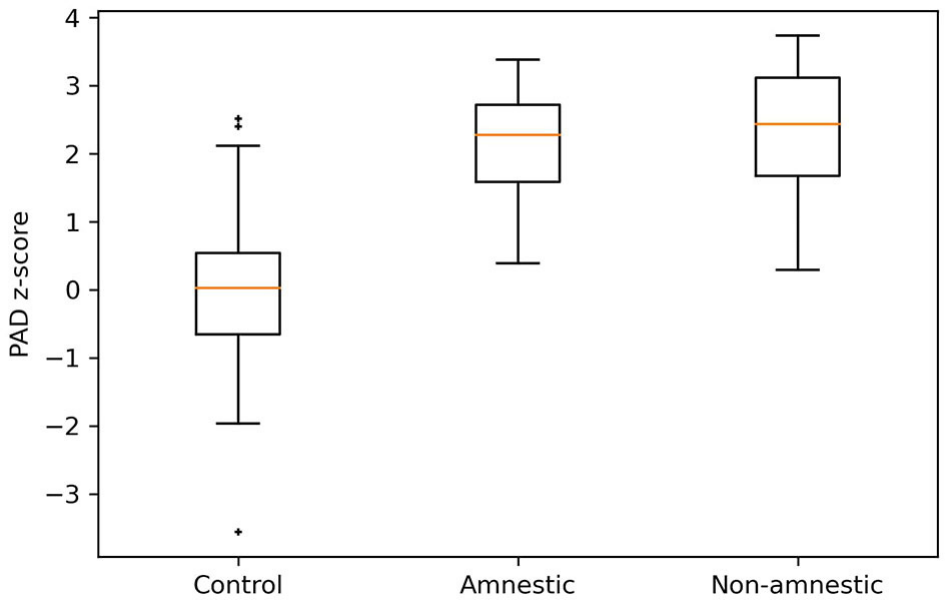
PAD z-scores distribution for controls, amnestic, and non-amnestic EOAD patients. A Kruskal–Wallis test was applied to these groups with a *p* < 0.001. Using Cliff's delta, we showed that there was an effect size between controls and non-amnestic population (*d* = 0.91), controls and amnestic population (*d* = 0.90), and non-amnestic and amnestic population (*d* = 0.22). PAD, predicted age difference. †, ‡; represent the outliers of the distribution.

Predicted age difference z-scores were different for the three groups (*p* < 0.01), with higher PAD z-scores for non-amnestic patients (2.35 ± 0.91) when compared to amnestic patients (2.09 ± 0.74, *p* = 0.022) and controls (0.00 ± 1, *p* < 0.001) (Figure 5). PAD z-scores were higher for amnestic patients when compared to controls (*p* < 0.001) (Figure 5). There was a small effect size between amnestic patients and non-amnestic patients (Cliff's delta = 0.22). For controls, there was a large effect size with amnestic (Cliff's delta = 0.90) and non-amnestic (Cliff's delta = 0.91) population.

**Figure 6:**
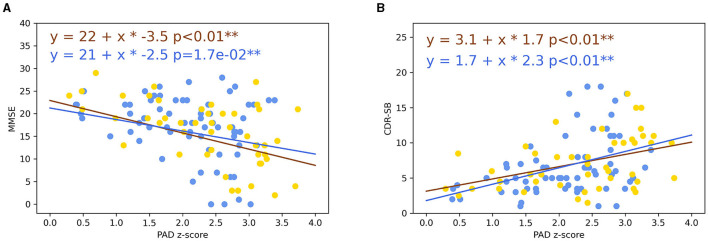
**(A)** Plot of the MMSE in function of PAD in the EOAD population. **(B)** Plot of the CDR-SB in function of PAD in the EOAD population. Lines represented linear trajectories using linear regression model (blue line: amnestic EOAD and yellow line: non-amnestic EOAD). **p < 0.05. MMSE, Mini-Mental State Examination; PAD, predicted age difference; CDR-SB, Clinical Dementia Rating Sum of Boxes; EOAD, early-onset Alzheimer's disease.

Predicted age difference z-scores were predictive of a low MMSE (non-amnestic patients: α = −3.5, intercept = 22, *p* < 0.001; amnestic patients: α = −2.5, intercept = 21, *p* = 1.7e-02) and a high CDR-SB score (non-amnestic patients: α = 1.7, intercept = 3.1, *p* < 0.001; amnestic patients: α = 2.3, intercept = 1.8, *p* < 0.001) for EOAD participants (Figure 6).

**Figure 7:**
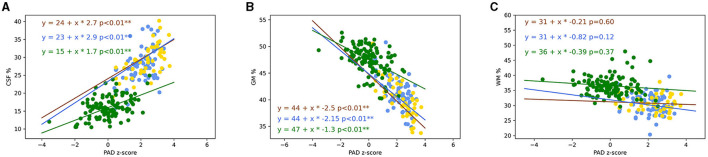
Plot of the percentage of brain CSF **(A)**, GM **(B)**, and WM **(C)** volumes in function of PAD. Lines represented trajectories using linear regression model (blue line: amnestic EOAD; yellow line: non-amnestic EOAD; and green line: control). Pearson correlation was computed between PAD and MMSE on non-amnestic and amnestic EOAD populations. The two coefficients of the regression were not statistically different between non-amnestic and amnestic compared to GM and CSF volumes (Chow test). The two coefficients of the regression for PAD in function of GM and CSF volumes were statistically different between controls and EOAD subtypes (Chow test). **p < 0.05. CSF, cerebrospinal fluid; PAD, predicted age difference; GM, gray matter; WM, white matter; EOAD, early-onset Alzheimer's disease; MMSE, Mini-Mental State Examination.

The percentage of CSF brain volume was positively correlated to PAD z-score for non-amnestic EOAD (*r* = 0.35, *p* = 0.0025), amnestic EOAD (*r* = 0.35, *p* = 0.0025), and control participants (r = 0.30, *p* < 0.001) (Figure 7A). Amnestic (*p* < 0.001) and non-amnestic EOAD patients (*p* < 0.001) had a more rapid evolution of PAD z-scores in relation to the percentage of CSF brain volume when compared to control participants (Figure 7A). Amnestic and non-amnestic EOAD patients had no statistical difference between PAD z-scores and percentage of CSF brain volumes (*p* = 0.1).

The percentages of GM volume were strongly negatively correlated to PAD z-scores for non-amnestic EOAD (*r* = −0.72, *p* < 0.001), amnestic EOAD (*r* = −0.55, *p* < 0.001), and control participants (*r* = −0.50, *p* < 0.001) (Figure 7B). Amnestic (*p* < 0.001) and non-amnestic EOAD patients (*p* < 0.001) had a more rapid evolution of PAD in relation to the percentage of GM volume when compared to control participants (Figure 7B). Amnestic and non-amnestic patients had no statistical difference between PAD z-scores and percentage of GM volumes (*p* = 0.092).

The percentages of WM volume were not correlated to PAD z-scores for non-amnestic (*r* = 0.01, *p* = 0.94), amnestic EOAD (*r* = −0.01, *p* = 0.89), and control participants (*r* = 0.11, *p* = 0.22) (Figure 7C).

### Attention Map

**Figure 8:**
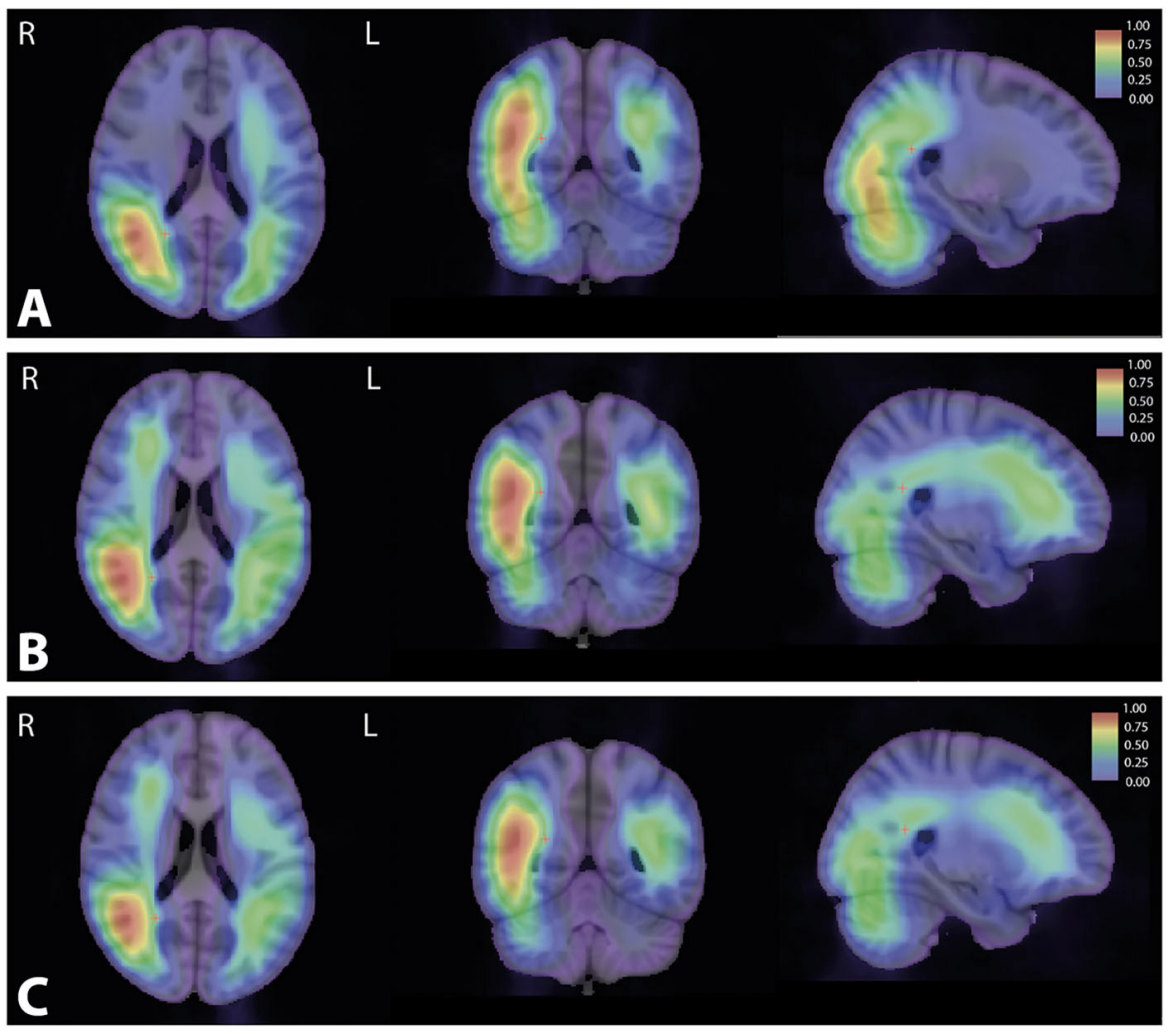
Attention maps computed by Grad-CAM for control, non-amnestic, and amnestic EOAD participants. The resulting averages for each population were overlaid on MNI template space. MNI coordinate of the red cross (25.28, −82.36, 33.42). **(A)** Average of the attention map on the control population. **(B)** Average of the attention map on the non-amnestic EOAD population. **(C)** Average of the attention map on the amnestic EOAD population. R, right hemisphere; L, left hemisphere; Grad-CAM, Gradient-weighted Class Activation Mapping; EOAD, early-onset Alzheimer's disease; MNI, Montreal Neurological Institute.

**Figure 9:**
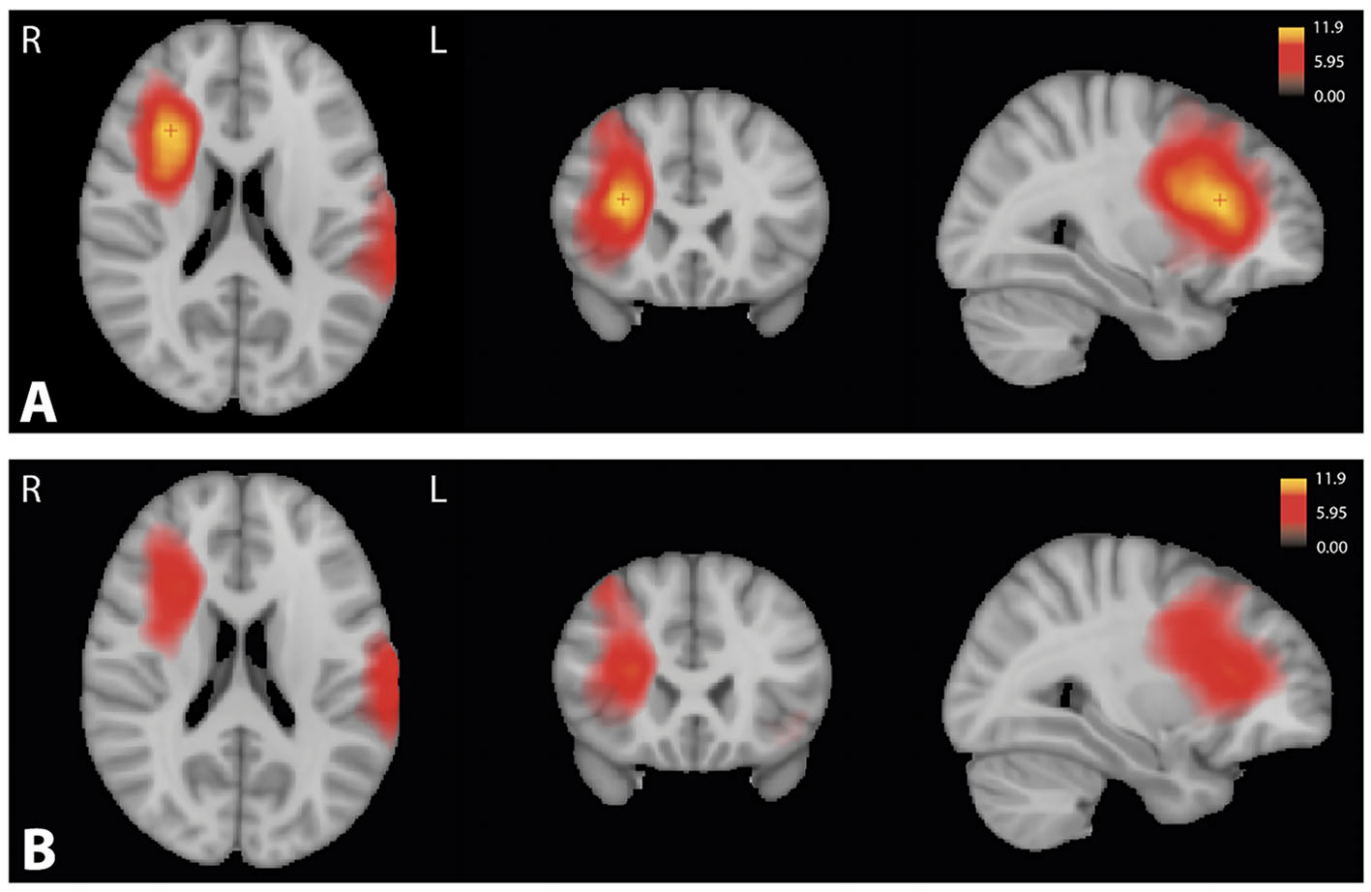
Significant *T*-value map (*p* < 0.05) computed using ANOVA corrected by the family-wise error method between the attention maps of the control group and **(A)** the amnestic and **(B)** the non-amnestic groups. Coordinate of the slice (25.11, 18.40, 25.92). R, right hemisphere; L, left hemisphere; ANOVA, Analysis of variance.

For all groups, attention maps showed that mostly subcortical white matter temporo-occipital junction and extension to subcortical white matter middle frontal gyrus were used for brain age prediction (Figures 8A–C). In these structures, we noticed a more important involvement of the right hemisphere. For amnestic and non-amnestic EOAD subtypes, attention maps were similar but statistically took into account more information than the controls (*p* < 0.05 FWE-corrected). EOAD subtypes had the involvement of the left superior temporal gyrus and right middle and inferior gyrus and anterior insula (Figures 9A,B).

### Longitudinal Analysis of Predicted Age Between EOAD Subtypes

**Figure 10:**
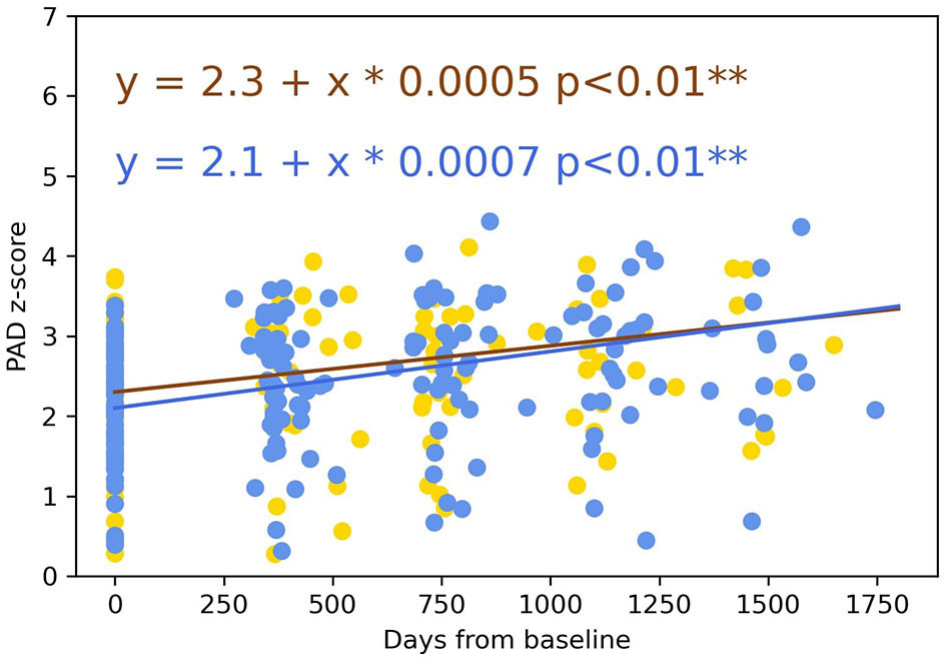
Longitudinal evolution of the PAD over 4 years between amnestic and non-amnestic patients. Lines represented trajectories using linear-mixed-effects models (yellow line: non-amnestic EOAD and blue line: amnestic EOAD). The two coefficients of the regression were not statistically different (Chow test, *p* = 0.096). PAD, predicted age difference; EOAD, early-onset Alzheimer's disease. **p < 0.05.

The gradual development of disease in patients was accompanied by a significant increase in PAD z-scores over time (*p* < 0.01) (Figure 10). Using the Chow test, there was no statistical difference (*p* = 0.096) observed in the PAD z-scores evolution between amnestic and non-amnestic EOAD patients.

## Discussion

In this study, we predicted the brain age of EOAD patients based on 3D-T1 images using a 3D CNN algorithm trained on a cohort of 1,955 healthy controls. We compared PAD z-scores in amnestic and non-amnestic EOAD patients and healthy controls. Although the groups of participants were matched with their chronological age, PAD z-scores were higher for EOAD patients when compared to controls. The brain regions used for brain age prediction were also different between groups with the involvement of left superior temporal gyrus and right middle and inferior gyrus and anterior insula in EOAD patients. Moreover, we showed that non-amnestic EOAD patients had a higher PAD z-score than amnestic EOAD patients. Finally, we compared PAD z-scores longitudinally over a period of 4 years and found that the PAD z-score increased with the severity of the disease.

The atrophied regions detected in AD patients largely overlapped with the regions showing a normal age-related decline in healthy control subjects (Raji et al., 2009). As PAD score was a way of quantifying an early brain age acceleration, it was an appropriate method to detect the development of neurodegenerative diseases such as AD. This characteristic of PAD score had already shown its potential to provide clinically relevant information (Franke et al., 2010). Studies showed that PAD score longitudinally increased with the severity of the impairment and allowed the detection of conversions from mild cognitive impairment to AD (Franke and Gaser, 2012; Gaser et al., 2013). However, these studies were based on the comparisons of AD and healthy controls, while several AD subtypes exist. In this study, we studied the EOAD which was defined by an age of onset ≤ 65 years. Due to its early onset and the clinical overlap between different diseases, there was a significant delay in the diagnosis of EOAD (van Vliet et al., 2013). One specificity of EOAD is the higher frequency of non-amnestic variants when compared to LOAD (Palasí et al., 2015). Amnestic and non-amnestic forms have different origins and evolution on the cerebral degeneration (Ossenkoppele et al., 2015; Xia et al., 2017; Phillips et al., 2018; Riedel et al., 2018; Vanhoutte et al., 2020). We investigated PAD score as a marker of phenotypic heterogeneity in EOAD for diagnosis.

At baseline, PAD z-scores were positively correlated with MMSE and negatively correlated with CDR-SB. A previous study showed similar results in AD (Beheshti et al., 2018). The correlations of PAD with CDR-SB and MMSE mean that PAD was correlated with the cognitive state of the patients. They confirmed that a more impaired patient tended to have a higher PAD. It was interesting to see that PAD was not only useful in separating healthy population from patients with neurodegenerative diseases, but that it was able to differentiate amnestic and non-amnestic EOAD patients (*p* = 0.022). Non-amnestic patients developed a more marked neocortical and basal nuclei atrophy. However, they had an identical severity on the MMSE, which could be due to a greater cognitive reserve because of a significantly higher level of education or due to the fact that the MMSE is less adapted to quantify the disorders in patients with executive type disorders. One of the strengths of our study is the fact that we analyzed the evolution of PAD z-scores between EOAD phenotypes over a period of 4 years. PAD z-scores increased over time similarly between amnestic and non-amnestic EOAD forms. This result corroborated the positive correlation between the severity of EOAD disease and the increase in PAD z-scores. The evolution of PAD z-scores over time was interesting because it could give a marker of evolution that was independent of the clinical form and of any other element such as cognitive scales that have their limits or clinical scales such as MMSE and CDR-SB (collected subjectively from the caregiver). This would make it possible to get rid of this heterogeneity and show the evolution in an objective way.

One originality of our study was the search for the interpretation of the PAD score in order to know about the brain information on which it was based. We computed the attention maps to show the most involved regions for the brain age prediction. For the three populations, the brain age model took into account common area of the brain with the involvement of the subcortical white matter temporo-parieto-occipital junction and the extension to subcortical white matter middle frontal gyrus. We noticed that the model took more information on the right hemisphere than on the left hemisphere. For the EOAD subtypes, the model statistically accounted for additional structures such as the left superior temporal gyrus and the right middle and inferior frontal gyrus and anterior insula. We also looked for elements of interpretation in the interactions of the PAD with the different tissues of CSF, WM, and GM, which changed throughout life. As GM tissues decreased with age (Narvacan et al., 2017), it was not surprising that PAD score was negatively correlated with the GM volume for each group. The GM volume did not decrease in the same way in all groups. EOAD had lower GM volume, which was consistent with the fact that AD experienced a faster decrease in GM volume when compared to aging subjects (Frings et al., 2014). Even if WM volume changed during the course of life (Guttmann et al., 1998; Courchesne et al., 2000), our brain age model did not use this information to compute PAD score. In aging, due to ventricular dilatation and the decrease of GM and WM volumes, the CSF volume increased with age (Courchesne et al., 2000). We observed a positive correlation between CSF brain volume and PAD score for each group. Nevertheless, EOAD patients had higher CSF brain volume than controls, which was in agreement with the previous studies on EOAD and LOAD patients (Anoop et al., 2010; Teng et al., 2014; Chiaravalloti et al., 2016; Falgàs et al., 2020b). The correlations were also consistent with the attention maps showing the involvement of the junction between the GM and the CSF in the brain age prediction.

To predict PAD score, we used a 3D CNN architecture which allowed us to work directly with raw data with few preprocessing steps. Using raw data allowed the algorithm to search itself for the available information regarding who was the most interesting to solve the brain age problem. There was therefore less bias induced by the preprocessing steps, which were usually more present during feature extraction for a classical machine learning algorithm. We applied field inhomogeneity correction, skull-stripped extraction, linear registration to common space, and min–max normalization as preprocessing steps. We obtained a cross-validation MAE of 3.18 ± 0.43 and a test MAE of 4.34, which are common results in brain age prediction (Franke and Gaser, 2019; Sajedi and Pardakhti, 2019). The requirement of few preprocessing steps and the fast calculation (around 0.36 s) make the PAD score a marker of sporadic EOAD subtypes classification that can be used in clinical routine.

Our study had some limitations. Even if 3,227 MR images are considered to be a great number in medical studies, CNN models used to be trained on a greater number of images. More complex brain aging models exist, but we made the choice to use a simpler and more flexible architecture to avoid overfitting. In addition, this brain age model had already proven its performance in a previous study (Cole et al., 2017a). Despite the correction of the regression toward mean, the problem was still present and was more pronounced when the model was used on different data from the training set. However, to the best of our knowledge, no alternative has been found to this problem apart from the regression of the error. Our population suffered from an attrition bias, as not all included patients completed the 4 years follow-up. The controls used for comparison to EOAD patients were not acquired with the same MR scanner. We selected healthy subjects not used during the implementation and validation of the brain age prediction model. Moreover, we randomly paired our control with our two EOAD subtypes based on age and sex, to remove the maximum of variability. Lastly, further independent validation will be necessary to assess the PAD score as a marker of global cognitive performance and clinical status.

## Conclusions

In this study, we showed that PAD score could be a valuable marker of disease severity which can be used to distinguish between clinical variants of EOAD. Further studies could determine the robustness of the PAD score in prospective cohorts and can be used in longitudinal studies for developments in pharmacological studies to show the arrest of this progression with treatment.

## Data Availability Statement

Publicly available datasets were analyzed in this study. This data can be found at: Information eXtraction from Images (https://brain-development.org/), Human Connectome Project (https://www.humanconnectome.org/), Center of Biomedical Research Excellence (https://www.mrn.org/common/cobre-phase-3), Mind Clinical Imaging Consortium (https://www.nitrc.org/projects/mcic/), Neuromorphometry by Computer Algorithm Chicago (http://schizconnect.org/), Enhanced Nathan Kline Institute-Rockland Sample (http://fcon_1000.projects.nitrc.org/indi/enhanced/), Parkinson's Progression Markers Initiative (www.ppmi-info.org/data), and Alzheimer's Disease Neuroimaging Initiative (adni.loni.usc.edu). Please find the accession numbers of the subjects used for training of the model in the file model_accession_numbers.csv and for the control population in the file control_accession_numbers.csv. (Supplementary Data Sheets 1, 2).

## Ethics Statement

The studies involving human participants were reviewed and approved by CPP Nord-Ouest I. The patients/participants provided their written informed consent to participate in this study.

## Author Contributions

MG: study design, MRI processing, statistical analyses, data interpretation, and writing of the manuscript. GK: study design, MRI processing, data interpretation, and critical revision of the manuscript. CB: acquisition and analysis of MRI data and critical revision of the manuscript. AS: acquisition of neuropsychological and clinical data. XD and ML: acquisition of neuropsychological data and critical revision of the manuscript. XL: critical revision of the manuscript. J-PP: study supervision and critical revision of the manuscript. FP: study supervision, acquisition of clinical data, and critical revision of the manuscript. RL: study concept, design and supervision, data interpretation, and critical revision of the manuscript. All authors contributed to the article and approved the submitted version.

## Funding

This research was funded by General Electric Healthcare as part of a scientific partnership with Lille University Hospital and the French government's LABEX DISTALZ program (development of innovative strategies for a transdisciplinary approach to Alzheimer's disease). Data collection and sharing for this project was funded by the Alzheimer's Disease Neuroimaging Initiative (ADNI) (National Institutes of Health Grant U01 AG024904) and DOD ADNI (Department of Defense Award Number W81XWH-12-2-0012). ADNI was funded by the National Institute on Aging, the National Institute of Biomedical Imaging and Bioengineering, and through generous contributions from the following: AbbVie, Alzheimer's Association; Alzheimer's Drug Discovery Foundation; Araclon Biotech; BioClinica, Inc.; Biogen; Bristol-Myers Squibb Company; CereSpir, Inc.; Cogstate; Eisai Inc.; Elan Pharmaceuticals, Inc.; Eli Lilly and Company; EuroImmun; F. Hoffmann-La Roche Ltd. and its affiliated company Genentech, Inc.; Fujirebio; GE Healthcare; IXICO Ltd.; Janssen Alzheimer's Immunotherapy Research & Development, LLC.; Johnson & Johnson Pharmaceutical Research & Development, LLC.; Lumosity; Lundbeck; Merck & Co., Inc.; Meso Scale Diagnostics, LLC.; NeuroRx Research; Neurotrack Technologies; Novartis Pharmaceuticals Corporation; Pfizer Inc.; Piramal Imaging; Servier; Takeda Pharmaceutical Company; and Transition Therapeutics. The Canadian Institutes of Health Research was providing funds to support ADNI clinical sites in Canada. Private sector contributions are facilitated by the Foundation for the National Institutes of Health (www.fnih.org). The grantee organization was the Northern California Institute for Research and Education, and the study was coordinated by the Alzheimer's Therapeutic Research Institute at the University of Southern California. ADNI data were disseminated by the Laboratory for Neuro Imaging at the University of Southern California. PPMI—a public–private partnership—was funded by the Michael J. Fox Foundation for Parkinson's Research and funding partners (the list of all of the PPMI funding partners can be found at: www.ppmi-info.org/fundingpartners). Data were provided by the Human Connectome Project, WU-Minn Consortium (Principal Investigators: David Van Essen and Kamil Ugurbil; 1U54MH091657) funded by the 16 NIH Institutes and Centers that support the NIH Blueprint for Neuroscience Research; and by the McDonnell Center for Systems Neuroscience at Washington University.

## Conflict of Interest

The authors declare that the research was conducted in the absence of any commercial or financial relationships that could be construed as a potential conflict of interest.

## Publisher's Note

All claims expressed in this article are solely those of the authors and do not necessarily represent those of their affiliated organizations, or those of the publisher, the editors and the reviewers. Any product that may be evaluated in this article, or claim that may be made by its manufacturer, is not guaranteed or endorsed by the publisher.
