# Supplementary material for: Longitudinal Analysis of Brain-Predicted Age in Amnestic and Non-amnestic Sporadic Early-Onset Alzheimer's Disease
Source: Front Aging Neurosci. 2021 Nov 3;13:729635. doi: 10.3389/fnagi.2021.729635 (PMC8596466; doi:10.3389/fnagi.2021.729635)
Supplement: Supplementary file 1 [file Table_1.DOCX]

Supplementary Material

# Supplementary Figures and Tables

| Information at baseline (M0) | M0 | | M12 | | M24 | | M36 | | M48 | |
| --- | --- | --- | --- | --- | --- | --- | --- | --- | --- | --- |
|  | Non-amnestic | Amnestic | Non-amnestic | Amnestic | Non-amnestic | Amnestic | Non-amnestic | Amnestic | Non-amnestic | Amnestic |
| N | 46 | 70 | 28 | 49 | 21 | 38 | 17 | 28 | 8 | 13 |
| CDR | 1.290.71 | 1.10.72 | 1.140.69 | 0.860.42 | 10.64 | 0.850.30 | 0.870.38 | 0.830.33 | 0.920.53 | 0.870.43 |
| MMSE | 16.136.96 | 17.146.71 | 18.925.99 | 19.364.48 | 19.095.2 | 19.473.77 | 21.234.33 | 20.603.21 | 22.872.90 | 21.153.60 |
| Disease duration, years | 4.552.14 | 5.452.91 | 3.791.93 | 5.272.61 | 4.051.85 | 4.992.45 | 3.401.49 | 4.932.10 | 3.261.60 | 4.302.30 |
| Education level, years | 10.412.82 | 9.642.82 | 10.212.88 | 9.852.92 | 9.852.76 | 9.342.54 | 10.822.55 | 9.753.39 | 11.372.87 | 9.764.02 |
| Age at baseline, years | 58.613.68 | 59.304.28 | 58.283.74 | 58.854.09 | 58.284.07 | 58.634.03 | 57.353.35 | 58.174.19 | 57.373.70 | 56.923.47 |
| Female, n (%) | 26(56%) | 36(51%) | 21 (53%) | 21 (42%) | 13 (61%) | 20 (50%) | 8 (47%) | 12 (42%) | 4 (50%) | 5 (38%) |

Table S1 - Information of the subjects at baseline for each year of our longitudinal acquisition.

| Study | n | Age, mean ±SD (years) | Age range, min-max (years) | Female, n (%) | Manufacturer | Website |
| --- | --- | --- | --- | --- | --- | --- |
| IXI | 181 | 46.87 ±16.73 | 20-81 | 94 (51%) | Philips | https://brain-development.org/ |
| HCP | 1783 | 28.76±3.7 | 22-37 | 968 (54%) | Siemens | https://www.humanconnectome.org/ |
| COBRE | 238 | 38.18±11.53 | 18-65 | 64 (26%) | Siemens | https://www.mrn.org/common/cobre-phase-3 |
| MCIC | 264 | 33.60±12.21 | 18-60 | 80 (30%) | Siemens | https://www.nitrc.org/projects/mcic/ |
| NmorphCH | 141 | 31.37±8.42 | 20-46 | 66 (46%) | Siemens | http://schizconnect.org/ |
| NKIRS | 620 | 42.62±18.27 | 18-85 | 406 (65%) | Siemens | http://fcon_1000.projects.nitrc.org/indi/enhanced/ |
| PPMI | 90 | 58.67±4.33 | 50-68 | 41 (45%) | General Electric / Philips / Siemens | https://www.ppmi-info.org/about-ppmi/ |
| ADNI | 27 | 60±2.6 | 57-66 | 22 (81%) | Philips / Siemens | http://adni.loni.usc.edu/ |

Table S2 - Informations about the subject for the model training
